# Supplementary material for: Thermally enhanced photoluminescence for heat harvesting in photovoltaics
Source: Nat Commun. 2016 Oct 20;7:13167. doi: 10.1038/ncomms13167 (PMC5080438; doi:10.1038/ncomms13167)
Supplement: Supplementary Information — Supplementary Figures 1-4, Supplementary Notes 1-5, Supplementary References. [file ncomms13167-s1.pdf]

Supplementary Information

Supplementary Figure 1

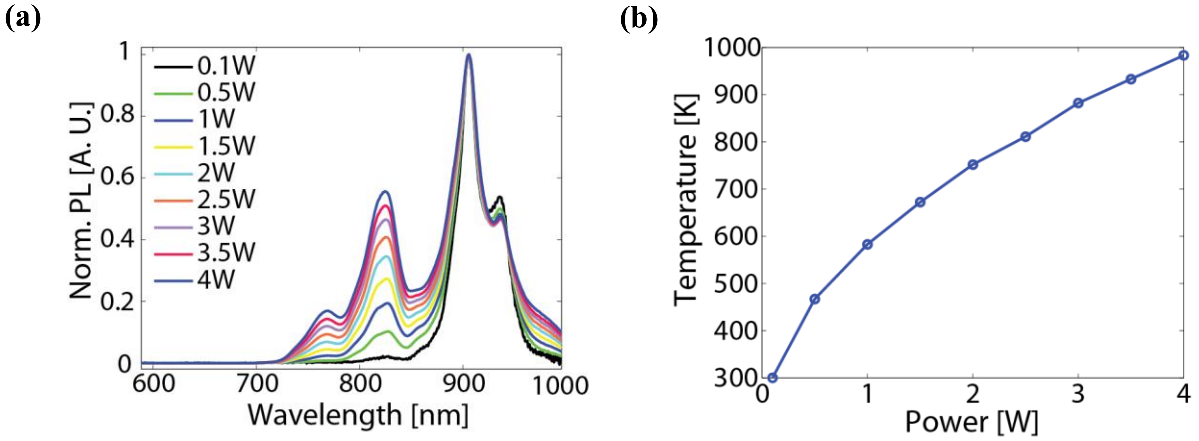

Temperature measurement of Nd:SiO_2. (a) The evolution of PL spectrum with input power (b) The corresponding sample temperature.

Supplementary Figure 2

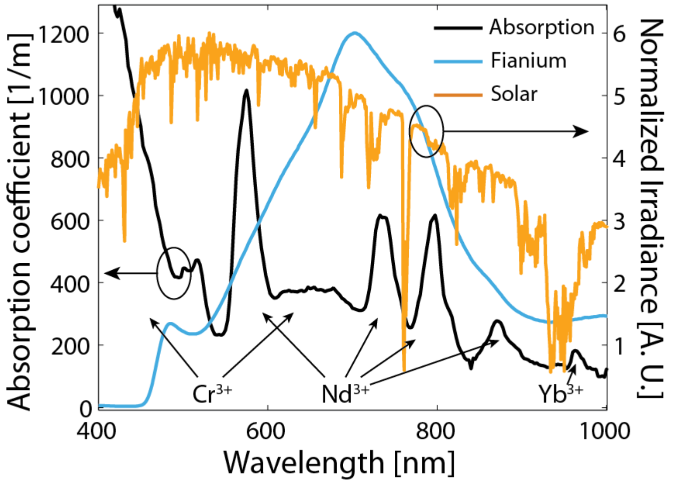

The glass absorption spectrum (black) and the white light excitation spectrum (blue). The solar spectrum is presented in orange for comparison.

Supplementary Figure 3

(a) (b)

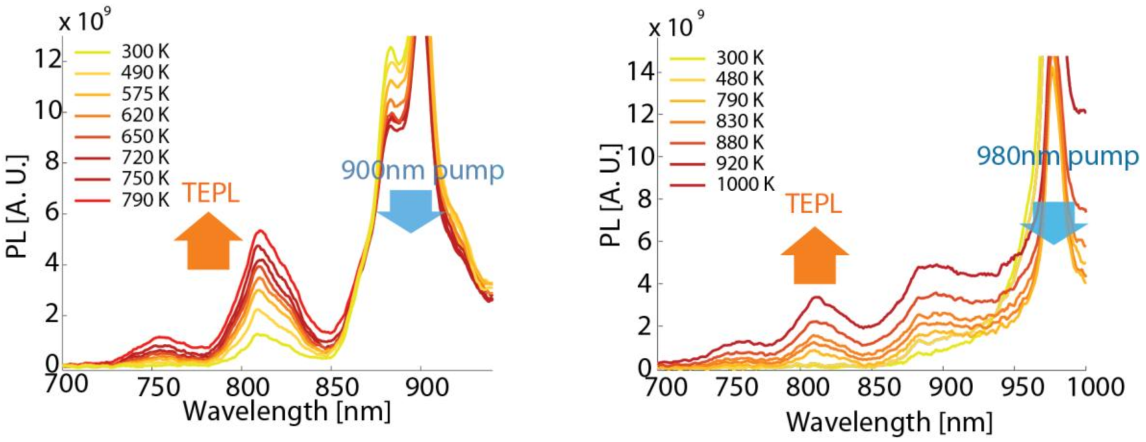

TEPL conversion of the 900nm (a) and 980nm (b) pumps upon CO_2 laser heating.

Supplementary Figure 4

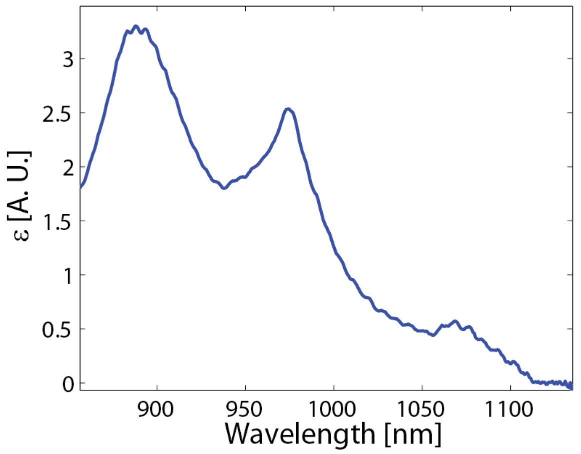

The sample emissivity (measured in A. U.) showing the rise in 1064 nm emissivity at high temperature (1600 K)

Supplementary Note 1

The detailed balance calculation. For the ideal case, we assume perfect absorption of photons above the bandgap, zero absorption of sub bandgap photons and unity EQE of both the absorber and the PV.

The PL photon rate R is given by the generalized Planck's law:

$$R(\hbar\omega, T, \mu) = \varepsilon(\hbar\omega) \cdot \frac{(\hbar\omega)^2}{4\pi^2\hbar^3c^2} \frac{1}{e^{\frac{\hbar\omega-\mu}{k_BT}}-1} \cong R_0(\hbar\omega, T) \cdot e^{\frac{\mu}{k_BT}} \qquad \text{Supplementary Equation (1)}$$

where R is the emitted photon flux (photons per second per unit area and solid angle, per energy interval $\hbar\omega$), T is the temperature, ε is the emissivity, $\hbar\omega$ is the photon energy, c is the speed of light in vacuum, k_b is Boltzmann's constant and $R_0(\hbar\omega, T)$ is the thermal emission rate. The corresponding emitted energy rate is defined by $E = \hbar\omega \cdot R(\hbar\omega, T, \mu)$. In our calculation, the emissivity is taken as a step function which corresponds to the material's band gap, i.e. zero below E_g and unity above it. In steady state, the balance of photon flux has to be satisfied - the incoming rate of absorbed photons is balanced by the emitted PL photon rate, which is either harvested by the PV or emitted towards the solid angle extended by the (concentrated) sun. In addition to the absorbed solar photons, The PV cell emits voltage-dependent PL towards the absorber, with an external efficiency $EQE_{PL,PV}$. Sub-bandgap photons are recycled back to the absorber with an efficiency *PR*. For the ideal case, we assume *PR*=100% (sub-bandgap photons are completely reflected by the PV back-reflector). Non-ideal *EQE* and *PR* values are taken into account in the non-ideal model, as described in section E.

Upon energetic-photon absorption, thermalization of electrons to the band-edge is accompanied by phonon emission. After the fast thermalization process (10^{-12} sec), the electrons are in thermal equilibrium with the lattice phonons at temperature *T*. The detailed balance is calculated for

changing absorber temperatures from 300 K up to 2000 K. For each temperature, the absorber's rate equation is solved to yields the absorber's chemical potential μ:

$$EQE\cdot\left[X\cdot\Omega_{\mathrm{sun}}\cdot\int_{E_{\mathrm{g,Abs}}}^{\infty}\overbrace{R_{\mathrm{sun,AM1.5}}\,\mathrm{d}\hbar\omega}^{\text{from sun}}+(2\pi-X\cdot\Omega_{sun})\cdot EQE_{\mathrm{PL,PV}}\int_{E_{\mathrm{g,PV}}}^{\infty}\overbrace{R_{\mathrm{PV}}(300,\mathrm{V})\,\mathrm{d}\hbar\omega}^{\text{from PV}}\right]=$$

$$(2\pi-X\cdot\Omega_{\mathrm{sun}})\cdot\left[\int_{E_{\mathrm{g,PV}}}^{\infty}\overbrace{R_{\mathrm{Abs}}(\mathrm{T},\mu)\,\mathrm{d}\hbar\omega}^{\text{to PV (above-band)}}+(1-\mathrm{PR})\cdot\int_{E_{\mathrm{g,Abs}}}^{E_{\mathrm{g,PV}}}\overbrace{R_{\mathrm{Abs}}(\mathrm{T},\mu)\,\mathrm{d}\hbar\omega}^{\text{PR loss (sub-band)}}\right]$$

$$+X\cdot\Omega_{\mathrm{sun}}\cdot\int_{E_{\mathrm{g,Abs}}}^{\infty}\overbrace{R_{\mathrm{Abs}}(\mathrm{T},\mu)\,\mathrm{d}\hbar\omega}^{\text{to sun}}$$

Supplementary Equation (2)

Where the left-hand side contains the incoming fluxes: X is the concentration ratio, Ω_{sun} is the solar solid angle, $R_{\mathrm{sun,AM1.5}}$ is the direct AM 1.5 solar spectrum, $EQE_{\mathrm{PL,PV}}$ is the PV cell PL *EQE* and V is the PV voltage. The right-hand side contains the emitted fluxes, where only above-bandgap PL is harvested by the PV.

Once $\mu(V)$ is known, the PV's detailed balance is made. The flux of absorbed photons from the absorber is balanced by the current extraction and the PV's spontaneous emission, to yield the system's current I:

$$(2\pi-X\cdot\Omega_{\mathrm{sun}})\cdot\int_{E_{\mathrm{g,PV}}}^{\infty}\overbrace{R_{\mathrm{Abs}}(\mathrm{T},\mu)\,\mathrm{d}\hbar\omega}^{\text{from absorber}}=(2\pi-X\cdot\Omega_{\mathrm{sun}})\cdot\int_{E_{\mathrm{g,PV}}}^{\infty}\overbrace{R_{\mathrm{PV}}(300,\mathrm{V})\,\mathrm{d}\hbar\omega}^{\text{to absorber}}+\frac{I}{e}$$

Supplementary Equation (3)

Where e is the elementary charge. Once the *I-V* curve is known, the efficiency is deduced by finding the maximal power point (MPP).

After the system is solved for every T, the solution is recalculated to find the total emitted energy by $E=\hbar\omega\cdot R(\hbar\omega,T,\mu)$, and validating it is smaller or equal to the total absorbed energy in order not to violate energy conservation.

Supplementary Note 2

The Nd: SiO_2 power spectrum measurements at *1000 K*. In addition to the presented experiment where a bulk of Nd^{3+} doped -Silicate glass was used to drive the PV at *600 K*, it is important to explore the concept in temperatures closer to the optimal of *1000 K*. As in Ref. 1, we pump a 50μ diameter Nd^{3+}:SiO_2 fiber tip. Such a small sample is insufficient to drive the PV when placed in an integrating sphere, but the power spectrum measurements capsulate all the required information about the predicted efficiency.

For this purpose, we test the maximal temperature that can be achieved with this system, without PL degradation. Supplementary Figure 1a shows the PL spectra of the sample when illuminated in vacuum with the high-power 532 nm laser. The FIR analysis (Supplementary Figure 1b) shows that the maximal temperature reached is ~*1000 K*, without significant thermal degradation of the PL signal. Since at higher temperatures a bigger portion of the PL signal is blue-shifted, the up-conversion efficiency is ideally monotonically rising with temperatures. When the temperature rises from *T=600 K* to *T=1000 K*, this portion increases by a factor of 2.6. We use this in order to deduce the maximal ideal obtainable conversion efficiency of the up-conversion experiment to be 6.7%.

Supplementary Note 3

The white-light TEPL experiment. As specified at the methods section, we fabricated a Cr:Nd:Yb doped glass absorber, which is tailored to continuously harvest the solar spectrum in the VIS-NIR range. The absorber's absorption spectrum is depicted in Supplementary Figure 2 (black line). The absorbing dopants which are responsible for each absorption line are marked. The excitation spectrum (Fianium) is shown in blue, compared to the solar spectrum in orange. We note the relatively low energetic photon content (for $\lambda<600\ nm$) of the pump in comparison to the Solar spectrum.

The white-light experiment consists of two parts. First, we verify TEPL conversion from the absorption lines at 900 nm (Nd^{3+}), 980 nm (Yb^{3+}), and 1064 nm (Nd^{3+}) composing the sub-band range (850 nm<λ<1000 nm). Here, in addition to the pumps, we use an additional CO_2 laser for the sample's heating. Supplementary Figure 3 shows the spectral evolution of the 900 nm and 980 nm pumps TEPL, upon heating. (Indicated by orange arrows). While the 900 nm TEPL process is originated only in the Nd^{3+}, in the 980 nm pump TEPL process photons are absorbed by the Yb^{3+} ground level and excite the $2F_{5/2}$ level. This is followed by sensitization of the Nd^{3+} $4F_{3/2}$ level which experiences TEPL conversion (Process shown in Figure 6a).

We also measure the sample's emissivity at high temperatures (which, according to Kirchoff's laws, is equal to the absorptivity) in order to deduce the high-temperature absorption coefficient of the 1064 nm transition. This is done by heating the sample with a CO_2 laser and deducing the sample's temperature by the FIR method. When the sample's temperature is known, the thermal emission is divided by the black-body function $I_{\lambda}(T)$ to obtain the emissivity lineshape (shown in A.U.). The emissivity calculated at T=1600 K is depicted in Supplementary

Figure 4, showing the rise in the 1064 nm line emissivity at high temperature. We then correct the room-temperature absorption spectrum, by the corrected ratio of the 1064nm/980nm peaks.

Supplementary Note 4

The practical TEPL device model. The model consists of the sunlight source, the absorber and PV cells as shown in figure 7a. This model assumes absorber temperature uniformity and neglects effect of non-uniform PV excitation on its efficiency. The ratio of energetic photons, $r_{energetic}(T)$, emitted above the bandgap of the PV to the total emission from the absorber, measured and displayed in figure 6d, is used as the basis of this model. The solar irradiation is taken to be AM1.5 and the absorption spectrum is as measured previously (Supplementary Figure 2). The ratio of the emission lost to the sun was assumed to be the ratio of the top facet area of the absorber out of the total surface area. For a cylinder this is $r_{lost} = D/(D+L)$ where the diameter D is chosen to be *3 mm* (assuring minimal transvers self-absorption), and the length L of 4.5cm is chosen to provide 98% absorption of sunlight. Eventually the emission of above-band photons is:

$$R_{\text{emission}} = R_{\text{absorbed}} \cdot \left(1 - r_{\text{lost}}\right) \cdot EQE \cdot \left(r_{\text{energetic}}(T) + PR \cdot \left(1 - r_{\text{energetic}}\right)\right) \qquad \text{Supplementary Equation (4)}$$

Where the *EQE* is the ratio of the number of emitted photons to the absorbed photon number, and the *PR* is the sub-bandgap photon recycling efficiency (when set to unity, sub-bandgap photons are ideally reflected by the PV back-reflector). The area of the PV cell, illuminated by this emission, is equal to the cylinder periphery area. To find the efficiency we calculate the expected current and the PV *I-V* curve by detailed balance. The efficiency is then calculated as the ratio of the maximal power point to the power incident from the sun.

Supplementary Note 5

The Fluorescence Intensity Ratio Thermometry (FIR) method. Using the FIR method, temperature can be measured by recording the intensity ratio of two adjacent optical emission peaks of a luminescent sample. Under Boltzmann population statistics, the ratio is defined by:

$$R = C \cdot e^{(-\frac{\Delta E}{k_B T})},$$

where ΔE is the energy difference between the two emission peaks. The constant is determined by the density of states and the matrix element for optical transitions between the states. Once ***C*** is known, the relation can be used to determine the sample's temperature during the experiment. In order to find *C*, we place the glass sample in a calibrated micro-furnace, which consists of a heated Quartz cylinder. The sample is pumped by a 532 nm laser, and the emitted PL signal is collected and focused onto a fiber-coupled spectrometer. The ratio *R* between the 900 nm and 820 nm peaks of the PL is measured as a function of the furnace temperature. The constant *C* is then calculated to yield a closed term for *R*, which enables temperature calculation from any given PL spectrum. (See reference 1 for more details).

Supplementary References

1. Manor, A., Martin, L. & Rotschild, C. Conservation of photon rate in endothermic photoluminescence and its transition to thermal emission. *Optica* **2,** 585 (2015).
